# 3-Year-Old Children Selectively Generalize Object Functions Following a Demonstration from a Linguistic In-group Member: Evidence from the Phenomenon of Scale Error

**DOI:** 10.3389/fpsyg.2016.00963

**Published:** 2016-06-24

**Authors:** Katalin Oláh, Fruzsina Elekes, Réka Pető, Krisztina Peres, Ildikó Király

**Affiliations:** ^1^Department of Cognitive Psychology, Institute of Psychology, Eötvös Loránd University, BudapestHungary; ^2^Psychobiology Research Group, Institute of Cognitive Neuroscience and Psychology, Centre for Natural Sciences, Hungarian Acadamy of Sciences, BudapestHungary; ^3^Department of Cognitive Science, Central European University, BudapestHungary

**Keywords:** scale error, object function, social category, learning, language

## Abstract

The present study investigated 3-year-old children’s learning processes about object functions. We built on children’s tendency to commit scale errors with tools to explore whether they would selectively endorse object functions from a linguistic in-group over an out-group model. Participants (*n* = 37) were presented with different object sets, and a model speaking either in their native or a foreign language demonstrated how to use the presented tools. In the test phase, children received the object sets with two modifications: the original tool was replaced by one that was too big to achieve the goal but was otherwise identical, and another tool was added to the set that looked different but was appropriately scaled for goal attainment. Children in the *Native* language condition were significantly more likely to commit scale errors – that is, choose the over-sized tool – than children in the *Foreign* language condition (48 vs. 30%). We propose that these results provide insight into the characteristics of human-specific learning processes by showing that children are more likely to generalize object functions to a category of artifacts following a demonstration from an in-group member.

## Introduction

Differentiating between people who belong to our social group from those who do not contributes greatly to our success in social interactions. The ability to detect the boundaries of social categories is not only vital in case of intergroup conflict, but it also helps us govern our behavior in everyday situations, such as determining what language to choose as the form of communication, how to interpret the behavior of the other person, etc. For adults, the process of categorization seems effortless and inevitable when faced with social stimuli (e.g., Taylor et al., 1978). Research with pre-school children have repeatedly shown that category-based thinking, stereotyping and in-group favoritism appear quite early in development (Aboud and Skerry, 1984).

In the past decades, ample evidence has been accumulated to support the notion that the tendency to perceive the social world as made up of groups emerges in infancy, and that this capacity may constitute a special faculty of the human mind (Kinzler and Spelke, 2007). For example, infants already at three months of age seem to be able to differentiate between female and male faces (Quinn et al., 2002), and they prefer to look at faces belonging to their own racial group (Kelly et al., 2005). Importantly, despite the early emergence of the ability to perceive the differences between various social categories, results suggest that some cues of group membership take precedence over others (Kinzler et al., 2010). A growing body of evidence highlights the importance of language in the process of categorization and developing social preferences. For example, Kinzler and Spelke (2011) have shown that 10-month-old and 2.5-year-old children do not preferentially interact with a racial in-group person, but using the same paradigm, a clear preference was observed for a native speaker over a person speaking a foreign language (Kinzler et al., 2007). In another study directly comparing the relevance of race and language in children’s choices of friends, 5-year-olds were found to select people who were from a different race, but spoke with their native accent over people of the same race but speaking with a foreign accent (Kinzler et al., 2009).

For children, the importance of identifying members of the same social group lies – at least partly – in the information it may provide about the knowledgeability of the individual (Kinzler et al., 2012; Oláh et al., 2014). Language and accent supposedly prove to be such reliable cues because they usually mark the boundaries of broader cultural groups; therefore people speaking the same language likely share other aspects of cultural knowledge as well. Keeping track of the knowledge state of others is a key factor behind conducting successful social interactions with others, yet it has a special significance for infants and children who are just in the process of acquiring knowledge about the world. Children will be most successful in this endeavor if they can select trustworthy and knowledgeable informants, who will provide information that is valid and useful within the given social context. Language and accent can be good indicators for children whether someone is potentially a reliable teacher for them.

So far, a handful of studies have explored the significance of linguistic group membership in infants’ and children’s willingness to accept information from someone. Kinzler et al. (2012) have shown that 10-month-old infants extend the preference for native speakers to the objects they interact with. When given the possibility to choose from two toys previously introduced by a native and a non-native speaker, infants reliably choose the one introduced by the native model. Similarly, Shutts et al. (2009) showed that 12-month-old infants’ choices of food were influenced by the emotional reactions of linguistic in-groups, but not that of out-groups.

Moreover, Buttelmann et al. (2013) have shown that 14-month-old infants were more likely to imitate a sub-optimal means to achieve a goal following a demonstration by an in-group member than by an out-group member. However no selectivity was observed in endorsing the object preferences of different group members. A study by Howard et al. (2015) further extends our understanding of social category based learning processes by showing that 19-month-old children only took into account the group membership of the model in an imitation task when the demonstration was administered on screen, but not in the case of live modeling. Contrarily, 3-year-old children selectively imitated an in-group member regardless of the mode of the demonstration, showing that selectivity becomes stronger with age.

A relevant question concerns children’s learning about object functions, since humans’ habits in using artifacts have an inherently cultural aspect. While an artifact may be appropriate to bring about several different goals, a very specific function is usually assigned to them during production. Adults and older children have a strong propensity to define object categories by the intended function, known as the *design-stance* (Dennett, 1989). Casler and Kelemen (2005) have shown that the precursors of this can be found in children as young as 2 years of age. Children of this age seem to represent objects as existing for certain purposes and view this purpose as an intrinsic property of the given object (*the teleo-functional stance*) – though they cannot yet explicitly give explanations in terms of the design-stance. It follows from such a conceptualization of artifact functions that they are not strictly or exclusively determined by the physical properties of the object, but that there is a partly arbitrary or incidental element in the process of assigning functions to objects. This arbitrary component makes object functions variable across cultures. Thus, object functions constitute a part of our cultural knowledge (e.g., whether we use a fork or chopsticks for eating).

Another important quality of object functions is that they are generally causally opaque by simple observation (Csibra and Gergely, 2009), therefore novices must rely on culturally knowledgeable individuals to pass on information about the intended function. In this study, we build on the phenomenon of scale error to investigate whether children can flexibly modulate their learning processes in response to the cultural group membership of the person demonstrating the object function.

The term scale error refers to young children’s tendency to disregard the actual size of the object they are interacting with when the object category is familiar to them. As a consequence, for example, they may try to slide down a miniature slide or try to squeeze themselves into a matchbox sized car (DeLoache et al., 2004). DeLoache et al. (2004) have demonstrated this phenomenon in children aged 18–30 months in a free-play setting and suggest that it may stem from an inability to integrate information from distinct processes in visual perception and from a lack of inhibitory control. Specifically, when children encounter an object that activates the representation of a kind of object, an action plan is formed based on stored knowledge of the object category. This action plan, however, does not become inhibited by size information as it would in the case of adults or older children. DeLoache et al. (2004) propose that this may be due to the lack of integration of information processed by the ventral and dorsal visual stream (Milner and Goodale, 1995) or a dissociation between action planning and control (Glover, 2004). Since the study by DeLoache et al. (2004), a number of studies have confirmed the robustness of scale errors (e.g., Rosengren et al., 2009; Ware et al., 2010).

Casler et al. (2011) have demonstrated the same phenomenon in two-year-old children with instrumental tool-use in a structured setting. In this study, children were presented with novel and familiar object sets. In the first phase of the experiment, a model demonstrated how to use the tools to achieve certain goals. Afterwards, children received the object sets with one alteration: the original tool was replaced by one that was either too big or too small to efficiently bring the goal about. Additionally, they received a novel object that was appropriate for goal attainment, but had not been presented during the demonstration. Under such circumstances, 2-year-old children committed scale errors 31% of the time. Casler et al. (2011) argue that a proneness to committing the scale error may originate from the early emerging teleo-functional stance (Casler and Kelemen, 2005), that is, to view artifacts as existing to serve certain functions. As a consequence, the function of the tool is incorporated into the representation of the object kind and when the category representation becomes active, it inevitably activates the representation of the task the object is for.

Although committing scale errors seems to be a robust phenomenon that has been demonstrated in numerous studies, the occurrence rate of it seems to vary with age. However, results from different studies do not show a clear trend of decreasing or increasing occurrence rates with age. DeLoache et al. (2004) have found that among 18–30 month-old participants, the 20.5–24 month-old group was the most prone to scale errors (with making 1.3 scale errors on 3 object sets on average). On the other hand, Ware et al. (2006) found that when testing children between the ages of 16–24, 29–32, and 35–40 months, the latter group committed the most scale errors.

In this study, we build on the assumption that scale errors occur with tools due to the fact that function constitutes an inherent part of stored knowledge about object categories. We propose that this makes the phenomenon of scale error sensitive to the context of knowledge acquisition. Research suggest that learning about object kinds happens with the help of specialized learning mechanisms that allow the observer to efficiently gain information about a category of objects from a single demonstration (e.g., Futó et al., 2010; Butler and Markman, 2012; for a general description see the Natural Pedagogy Theory, Csibra and Gergely, 2006, 2009; Gergely and Csibra, 2006). Cues, such as eye-contact, specific intonation, and addressing prompt the learner to extract generalizable knowledge from the demonstration (as opposed to episodic information), thus contributing to the generation and enrichment of knowledge stored about object kinds. However, as described in the beginning of this review, efficient learning also requires an ability to select knowledgeable teachers, who can provide valid information. Therefore, we hypothesized that if tool functions were presented by in-group models, children would be more prone to subsequently committing scale errors since the demonstrated function would be more likely to be incorporated into the representation of the object. We followed the methods of Casler et al. (2011) with the modification that the demonstrator was either presented as a speaker of children’s native language or a foreign language. We involved 3-year-old children in the study, as this is the age where both the occurrence of scale errors (e.g., Ware et al., 2006) and selectivity based on the linguistic group membership of the model (Howard et al., 2015) have been robustly demonstrated.

## Materials and Methods

The study was carried out with the approval of the Research Ethics Committee of the Faculty of Education and Psychology of Eötvös Loránd University. Participants’ caregivers gave written informed consent.

### Participants

Participants were 37 monolingual Hungarian children (14 girls) recruited through advertisements in the local area. Their ages ranged from 30 to 40 months, with a mean of 33.31 months (*SD* = 2.69). Children were randomly assigned to either the *Native (n* = 17) or the *Foreign* (*n* = 20) language condition. An additional 9 children were tested but later excluded from the sample due to passivity (3), camera failure (3), experimenter error (2), and the child was bilingual (1).

### Materials

**FIGURE 1:**
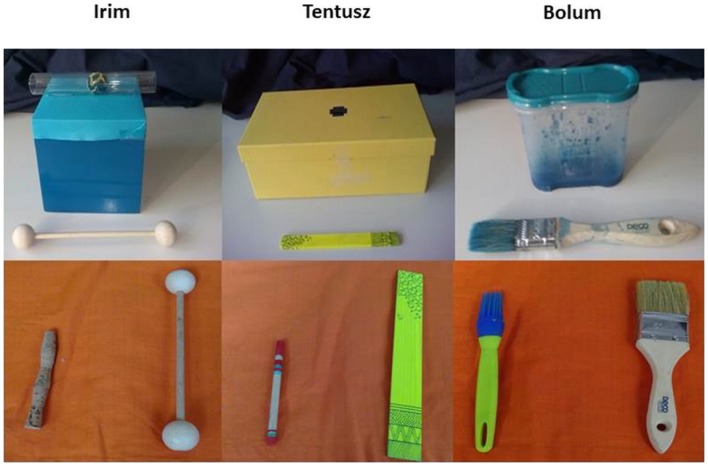
**Materials.** The picture depicts the target objects with the original tool and the corresponding over-sized and alternate tools presented in the test phase. The original tool was named by a non-word (see top row) in the beginning of each demonstration.

The object sets used in the study were inspired by three of the object sets used in the study of Casler et al. (2011). Each set consisted of a target object and three potential tools. There was one tool used in the demonstration phase and two presented in the test phase. One tool used in the test phase was identical to the one introduced during demonstration except that it was too big to bring about the goal, whereas the other testing tool was an alternate to the originally presented one with different perceptual features but corresponding size and affordances. The first object set could be used to paint on paper and consisted of a container with blue paint mixed with water inside (11 × 10.5 × 5 cm), a small paintbrush (19 cm long with a 3.5 × 1.5 cm head), a larger paintbrush that could not fit into the container (24 cm long with a 4.5 × 1.5 cm head) and a silicone brush (19 cm long with a 2.5 × 1.3 cm head). The second object set consisted of a yellow box (25.6 × 12.5 × 9.5) with a hole (1.5 × 1.2 cm) on top and a plastic toy inside that made a whistling noise when pushed on. The small tool used in the demonstration was a yellow wooden flat stick (14.9 cm long, 1.4 cm wide), while its larger counterpart was 29.7 × 3.9 cm in size. The alternate tool was a cylindrical stick painted red (14.8 cm, 9 mm diameter). The target action entailed inserting the tool in the hole to push on the toy inside the box and to elicit the sound. The last object set constituted of a blue box (10 × 10 × 10 cm) with a transparent tube (1.9 cm diameter) attached on top and a small ball inside the tube. The originally presented small tool was a thin wooden stick (14.5 cm long) with wooden balls (1.8 cm diameter) attached on both ends. Its larger counterpart was 25 cm long, while the balls attached were 3.5 cm in diameter. The alternate tool was a stick made out of cork (13 cm long, diameter: 1.5 cm). The target action entailed pushing the ball out of the tube with the help of the tool. For the object sets (see **Figure 1**).^1^

### Procedure

Experiments were conducted by 4 female experimenters of whom 2 took turns in taking the role of Experimenter 1 (E1) and 2 took turns in taking the role of Experimenter 2 (E2); however, the roles were counterbalanced across conditions, thus each experimenter participated in both the *Foreign* and the *Native* language conditions. E1 was the person greeting the participants and administering the test trials, while E2 played the role of the demonstrator. E1 always spoke in the child’s native language, whereas the language E2 used depended on condition.

Upon arrival to the laboratory, children were received by E1, who invited the child to participate in a session of free play in order to familiarize children with the environment and the experimenter. When the child seemed comfortable in the setting, E1 escorted the child and the caregiver into the testing room, where children were seated on the caregiver’s lap in front of a small table. E1 then told the child that she would be back in a few seconds and left. At this point, a second female experimenter (E2) entered the room and sat down at the opposite end of the table. She started the demonstration by saying three sentences either in Hungarian (participants’ native language) or in German (a foreign language to the participants). The sentences were construed in a way that they did not help the interpretation of the object function demonstration, but were not completely unrelated to the context in order to avoid confusing children in the *Native* condition. The sentences could be translated into English as follows: “Where have I put my things? They must be here somewhere. Ah, there they are!”. After that, she pulled out the first object set containing the target object and the small tool. She took the tool in her hand, looked at the child with a smile, named the tool by a non-word and demonstrated the action. Then, she put away the object set and performed the demonstration with the other two object sets one after the other. When the demonstration was over, E2 left the room and E1 re-entered. E1 sat down and said to the child: “Now let’s play something, shall we? Let me just see what we have here!” She then pulled out the first object set from behind a panel with two alterations compared to the initial demonstration. The tool used in the first phase was replaced by its larger counterpart that was inappropriately scaled to bring about the same goal. The alternate tool was also presented this time. The two tools were placed on the two sides of the target object. Children were allowed to interact with the object set for as long as they showed interest. After that, E1 put away the object set and presented the next one with the same alterations. All children received 3 trials, one with each object set. Children received the object sets in two pre-defined orders. The order and the side of the tools in the test phase were counterbalanced across conditions.

### Coding

We analyzed children’s choices of tools in the first 1.5 min of the interaction phase with each object set. Only children’s first choices were taken into account and we coded whether it was the over-sized (committing the scale error) or the alternate tool (not committing the scale error). Children not choosing a tool during this time were considered passive on the trial (object set) and the trial was excluded from analyses (*Native*: 2 out of 51 trials; *Foreign*: 6 out of 60 trials). An independent coder blind to the research question coded 80% of the videos. Reliability between the coders was good (Cohen’s kappa: 0.86).

To test whether children were equally attentive in the *Foreign* condition as in the *Native* condition, children’s looking behavior during the demonstration phase was also coded using Solomon Coder (András Péter).^2^ We coded the time children spent looking at each action demonstration from the moment E2 named the object she was about to use until the moment she started putting away the object set in question. We found that children in both conditions were attentive for almost the whole duration of the demonstrations (98.6% in the *Foreign* condition and 99.29% in the *Native* condition). The difference between conditions was not significant (*t*(30) = 0.72; *p* = 0.48).

## Results

Statistical analyses were performed with the SPSS 20 software. We used Generalized Linear Mixed Models (GLMM) with binary regression to test for differences in the occurrence of scale errors across conditions. We used this method for analyses since the dependent variable is not continuous, but is composed of three nominal values (a choice between the oversized tool and the novel tool on three trials). Therefore a GLMM is the best option as it can treat the different trials separately and thus provides a more elaborate test of the question. We used backwards modeling, where the following variables were included in the initial model, but were later removed as they were not significant: sex and age of the child, the presentation order of the object sets, side of the tools used in the test, the identities of the two experimenters, object type (novel/familiar). “Participant” was added to the model as a factor and “trial” as the repeated measure. In addition to these effects, only condition as a fixed effect was included in the final model.

**FIGURE 2:**
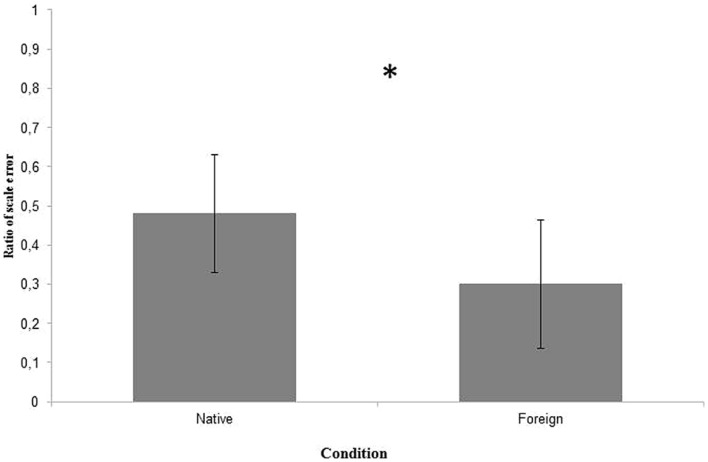
**Avarage ratio of scale errors committed in the Native and the Foreign language conditions on the three trials.** The asterisk indicates that the difference is significant at the level of 0.05.

Condition had a significant main effect on the amount of scale errors committed by children, with more scale errors occurring in the *Native* as opposed to the *Foreign* condition (*F* (1, 101) = 4.024; *p* = 0.048). On average, participants in the *Native* language condition committed the scale error on 48% of the trials, whereas the rate was 30% in the *Foreign* language condition (**Figure 2**).

The same effect of condition held with a simple comparison of proportions (occurrence rate of scale errors in the two conditions) using a χ^2^ test (χ^2^(1) = 6.81; *p* = 0.009).

## Discussion

Building on the phenomenon of scale error, the present study investigated whether 3-year-old children’s learning processes about tool functions would be influenced by the group membership of the person introducing the objects to them. We found that children were less prone to committing scale errors if the demonstration was performed by a person speaking in a foreign language. We propose that this result does not merely inform us about a quite specific phenomenon described in the developmental literature –that is, the occurrence of scale errors – but it reflects the special characteristics of human-specific learning mechanisms. As described in the introduction, scale errors supposedly occur because children do not treat the artifacts they encounter as individual and unique objects, but form representations of object kinds, during which the function assigned to the category of the artifact becomes a core characteristic (Casler et al., 2011). It has been suggested that a specialized learning mechanism helps children to extract kind-relevant, generalizable information from a single demonstration if the interactional partner expresses their intention of passing on knowledge (Csibra and Gergely, 2009). In this study, children supposedly committed the scale error on nearly half of the trials in the *Native* condition because they regarded the initial demonstration as an instance of teaching. Thus, the above-described genericity-bias (Csibra and Gergely, 2009) led them to retrieve the acquired knowledge (the function of the tool) in the presence of another exemplar of the same category (the over-sized counterpart of the original object) and children tried to enforce that function on the given exemplar irrespective of its actual size. It has also been shown that children are more prone to committing scale errors when the objects are named during the demonstration (Hunley and Hahn, 2016). This labeling effect with scale errors originates from the phenomenon that learning of object kinds is facilitated by naming the object (e.g., Booth and Waxman, 2002). In our study, this enhanced the proneness to committing scale errors in the *Native* condition, but children were not equally willing to accept the information from the model speaking a foreign language.

We thus propose that the decreased occurrence rate of scale errors in the *Foreign* language condition can be accounted for by the selectivity children exhibit in learning situations. That is, even though children may perceive the teaching intention exhibited by the model, a specific mistrust toward the epistemic state of the model leads them to refuse to endorse the information presented. Results suggest that even though pedagogical cues facilitate learning in general, young children are not equally willing to accept information from all sources. For example, 14-month-old infants are reluctant to imitate a model whose past behavior has turned out to be misleading (Poulin-Dubois et al., 2011) or could be seen as incompetent based on the level of confidence they exhibited (Zmyj et al., 2010). Importantly, studies have also shown that children show selectivity based on the language the potential teacher speaks (e.g., Buttelmann et al., 2013; Howard et al., 2015). Language cues can be of special importance when acquiring culturally relevant knowledge (such as tool functions) since the use of a foreign language is an indication that the person is not familiar with the ways of the given culture. Consequently children may not view them as a reliable source of information. We propose that the drop in the occurrence of scale errors reflect children’s resistance to accept the foreign language speaking model as a teacher and therefore they did not extract kind-based knowledge about the objects, which subsequently led to less confusion in the test phase.

Alternatively, the decreased occurrence of scale errors in the *Foreign* condition may not reflect a mistrust in the model, but a failure to encode the teaching intentions of a foreign speaker. This could possibly be the result of an intuition that members of different cultural groups keep to different norms in their behavior; therefore children could exhibit more confusion when interpreting the signals of an out-group member. While this interpretation is not perfectly independent of the one outlined in the previous section, nor are the two necessarily mutually exclusive, the sensitivity to communicative cues may constitute such a fundamental and universal capacity of the human mind (Csibra and Gergely, 2011), that it is unlikely to be disrupted in such a case.

An alternative explanation for our result could be that children simply paid less attention to the foreign language model and that is why scale errors occurred with less frequency. However, this explanation is not likely, as children were equally attentive during the demonstration regardless of condition and they seemed to understand the basic structures of the different tasks (they attempted to achieve the goal that was demonstrated). If children had been simply inattentive in the *Foreign* condition, then we would have expected to see instances where children were simply lost at how to interact with the novel objects. However, this was not the case; children reached for one of the tools on almost all trials in both conditions (see the section on *Coding*).

Altogether, our participants committed more scale errors than children in the study of Casler et al. (2011). While the ratio of scale errors in their study is comparable to that found in the Foreign language condition (around 30%), this number was substantially higher in the Native language condition (48%). This may be accounted for by the fact that we did not use the exact same object sets as did Casler et al. (2011). Instead of using four object sets, we settled on replicas of three of the ones used in their study. Importantly, Casler et al. (2011) have found that children committed the most scale errors when presented with novel apparatuses and novel tools (40%). In our study, two out of three object sets can be regarded as novel tool-novel apparatus sets, which could have resulted in higher overall ratios.

On the one hand, our study contributes to our understanding of how the group membership of the model influences children’s learning processes. On the other, it provides a further piece of evidence to support the claim that scale errors cannot solely be explained in terms of problems of inhibitory control, but that they result from the way children form representations of tools and their functions (Casler et al., 2011). Specifically, children view artifacts as being for certain functions and they treat this artifact-function correspondence in quite a rigid way. Furthermore, our study suggests that scale errors are at least partly the result of the characteristics of human-specific learning processes that result in viewing an object as having a fixed function.

## Author Contributions

KO, FE, and IK conceived the experiment; KO, PR, and KP ran the experiments; KO, RP and IK analyzed tha data; KO prepared the first draft of the manuscript and FE, RP, KP, and IK provided feedback. The final version of the manuscript was submitted with the approval of all of the authors.

## Conflict of Interest Statement

The authors declare that the research was conducted in the absence of any commercial or financial relationships that could be construed as a potential conflict of interest.

## Footnotes

1 The object sets could be categorized as either completely novel to children (the yellow box with the toy inside and the blue box with the tube on top) or familiar (painting).

2 http://solomoncoder.com

## References

[B1] Aboud F. E. Skerry S. A. (1984). The development of ethnic attitudes a critical review. *J. Cross Cult. Psychol.* 15 3–34. 10.1177/0022002184015001001

[B2] Booth A. E. Waxman S. (2002). Object names and object functions serve as cues to categories for infants. *Dev. Psychol.* 38 948 10.1037/0012-1649.38.6.948 12428706

[B3] Butler L. P. Markman E. M. (2012). Preschoolers use intentional and pedagogical cues to guide inductive inferences and exploration. *Child Dev.* 83 1416–1428. 10.1111/j.1467-8624.2012.01775.x 22540939

[B4] Buttelmann D. Zmyj N. Daum M. Carpenter M. (2013). Selective imitation of in-group over out-group members in 14-month-old infants. *Child Dev.* 84 422–428. 10.1111/j.1467-8624.2012.01860.x 23006251

[B5] Casler K. Eshleman A. Greene K. Terziyan T. (2011). Children’s scale errors with tools. *Develop. psychol.* 47 857 10.1037/a0021174 21142360

[B6] Casler K. Kelemen D. (2005). Young children’s rapid learning about artifacts. *Develop. sci.* 8 472–480. 10.1111/j.1467-7687.2005.00438.x 16246238

[B7] Csibra G. Gergely G. (2006). Social learning and social cognition: the case for pedagogy. *Process. Change Brain Cogn. Dev. Attent. Perform. XXI* 21 249–274.

[B8] Csibra G. Gergely G. (2009). Natural pedagogy. *Trends cogn. Sci.* 13 148–153. 10.1016/j.tics.2009.01.005 19285912

[B9] Csibra G. Gergely G. (2011). Natural pedagogy as evolutionary adaptation. *Philos. Trans. R. Soc. Lond. B Biol. Sci.* 366 1149–1157. 10.1098/rstb.2010.0319 21357237 PMC3049090

[B10] DeLoache J. S. Uttal D. H. Rosengren K. S. (2004). Scale errors offer evidence for a perception-action dissociation early in life. *Science* 304 1027–1029. 10.1126/science.1093567 15143286

[B11] Dennett D. C. (1989). *The intentional stance.* Cambridge, MA: MIT press.

[B12] Futó J. Téglás E. Csibra G. Gergely G. (2010). Communicative function demonstration induces kind-based artifact representation in preverbal infants. *Cognition* 117 1–8. 10.1016/j.cognition.2010.06.003 20605019

[B13] Gergely G. Csibra G. (2006). “Sylvia’s recipe: The role of imitation and pedagogy in the transmission of cultural knowledge,” in *Roots of Human Sociality: Culture, Cognition, and Human Interaction* eds Enfield N. J. Levenson S. C. (Oxford: Berg) 229–255.

[B14] Glover S. (2004). Separate visual representations in the planning and control in action. *Behav. brain sci.* 27 3-24. 10.1017/S0140525X04000020 15481943

[B15] Howard L. H. Henderson A. M. Carrazza C. Woodward A. L. (2015). Infants’ and young children’s imitation of linguistic in-group and out-group informants. *Child dev.* 86 259–275. 10.1111/cdev.12299 25263528 PMC4358791

[B16] Hunley S. B. Hahn E. R. (2016). Labels affect preschoolers’ tool-based scale errors. *J. Exp. Child Psychol.* 10.1016/j.jecp.2016.01.007 [Epub ahead of print]. 26925720

[B17] Kelly D. J. Quinn P. C. Slater A. M. Lee K. Gibson A. Smith M. (2005). Three-month-olds, but not newborns, prefer own-race faces. *Dev. sci.* 8 F31–F36. 10.1111/j.1467-7687.2005.0434a.x 16246233 PMC2566511

[B18] Kinzler K. D. Dupoux E. Spelke E. S. (2007). The native language of social cognition. *Proc. Natl. Acad. Sci. U.S.A.* 104 12577–12580. 10.1073/pnas.0705345104 17640881 PMC1941511

[B19] Kinzler K. D. Dupoux E. Spelke E. S. (2012). ‘Native’objects and collaborators: infants’ object choices and acts of giving reflect favor for native over foreign speakers. *J. Cogn. Dev.* 13 67–81. 10.1080/15248372.2011.567200 23105918 PMC3478775

[B20] Kinzler K. D. Shutts K. Correll J. (2010). Priorities in social categories. *Eur. J. Soc. Psychol.* 40 581–592. 10.1002/ejsp.739

[B21] Kinzler K. D. Shutts K. DeJesus J. Spelke E. S. (2009). Accent trumps race in guiding children’s social preferences. *Soc. cogn.* 27 623 10.1521/soco.2009.27.4.623 PMC3096936 21603154

[B22] Kinzler K. D. Spelke E. S. (2007). Core systems in human cognition. *Prog. Brain Res.* 164 257–264. 10.1111/j.1467-7687.2007.00569.x 17920436

[B23] Kinzler K. D. Spelke E. S. (2011). Do infants show social preferences for people differing in race? *Cognition* 119 1–9. 10.1016/j.cognition.2010.10.019 21334605 PMC3081609

[B24] Milner A. D. Goodale M. A. (1995). *The Visual Brain in Action* Vol. 27 New York: Oxford University Press.

[B25] Oláh K. Elekes F. Bródy G. Király I. (2014). Social category formation is induced by cues of sharing knowledge in young children. *PLoS One* 9:e101680 10.1371/journal.pone.0101680 PMC4094464 25014363

[B26] Poulin-Dubois D. Brooker I. Polonia A. (2011). Infants prefer to imitate a reliable person. *Infant Behav. Dev.* 34 303–309. 10.1016/j.infbeh.2011.01.006 21353308

[B27] Quinn P. C. Yahr J. Kuhn A. Slater A. M. Pascalis O. (2002). Representation of the gender of human faces by infants: A preference for female. *Perception* 31 1109–1122. 10.1068/p3331 12375875

[B28] Rosengren K. S. Gutiérrez I. T. Anderson K. N. Schein S. S. (2009). Parental reports of children’s scale errors in everyday life. *Child Dev.* 80 1586–1591. 10.1111/j.1467-8624.2009.01355.x 19930339

[B29] Shutts K. Kinzler K. D. McKee C. B. Spelke E. S. (2009). Social information guides infants’ selection of foods. *J. Cogn. Dev* 10 1–17. 10.1080/15248370902966636 19809590 PMC2756712

[B30] Taylor S. E. Fiske S. T. Etcoff N. L. Ruderman A. J. (1978). Categorical and contextual bases of person memory and stereotyping. *J. Pers. Soc. Psychol.* 36 778 10.1037/0022-3514.36.7.778

[B31] Ware E. A. Uttal D. H. DeLoache J. S. (2010). Everyday scale errors. *Dev. Sci.* 13 28–36. 10.1111/j.1467-7687.2009.00853.x 20121860

[B32] Ware E. A. Uttal D. H. Wetter E. K. DeLoache J. S. (2006). Young children make scale errors when playing with dolls. *Dev. Sci.* 9 40–45. 10.1111/j.1467-7687.2005.00461.x 16445394

[B33] Zmyj N. Buttelmann D. Carpenter M. Daum M. M. (2010). The reliability of a model influences 14-month-olds’ imitation. *J. Exp. Child Psychol.* 106 208–220. 10.1111/j.1467-8624.2012.01860.x 20427052

